# Exploring the potential association between brominated diphenyl ethers, polychlorinated biphenyls, organochlorine pesticides, perfluorinated compounds, phthalates, and bisphenol a in polycystic ovary syndrome: a case–control study

**DOI:** 10.1186/1472-6823-14-86

**Published:** 2014-10-28

**Authors:** Sara J Vagi, Eduardo Azziz-Baumgartner, Andreas Sjödin, Antonia M Calafat, Daniel Dumesic, Leonardo Gonzalez, Kayoko Kato, Manori J Silva, Xiaoyun Ye, Ricardo Azziz

**Affiliations:** U.S. Centers for Disease Control and Prevention, Atlanta, GA USA; University of California, Los Angeles, CA USA; Cedars Sinai Medical Center, Los Angeles, CA USA; Georgia Regents University, 1120 15th St, Rm. AA-311, 30904 Augusta, GA USA

## Abstract

**Background:**

Polycystic Ovary Syndrome (PCOS) is an endocrine-metabolic disorder that affects approximately 6-10% of women of child-bearing age. Although preliminary studies suggest that certain pollutants may act as endocrine disruptors in animals, little is known about their potential association with PCOS. The objective of this case-control pilot study is to determine whether women with PCOS have higher concentrations of specific environmental contaminants compared to women who have not developed PCOS.

**Methods:**

Fifty-two PCOS case-patients (diagnosed using the National Institutes of Health 1990 definition) and 50 controls were recruited in 2007–2008, from an urban academic medical center in Los Angeles, CA. Brominated diphenyl ethers, polychlorinated biphenyls (PCBs), organochlorine pesticides, and perfluorinated compounds (PFCs) were measured in serum, and phthalates metabolites and bisphenol A (BPA) in urine.

**Results:**

PCOS case-patients had significantly higher geometric mean (GM) serum concentrations of two PFCs: perfluorooctanoate (PFOA) (GM_cases_ = 4.1 μg/L, GM_controls_ = 2.3 μg/L; p = 0.001) and perfluorooctane sulfonate (PFOS) (GM_cases_ = 8.2 μg/L, GM_controls_ = 4.9 μg/L; p = 0.01), and lower urinary concentrations of monobenzyl phthalate (mBzP) (GM_cases_ = 7.5 μg/g creatinine, GM_controls_ = 11.7 μg/g creatinine; p = 0.02). Logistic regression, controlling for body mass index, age and race, identified an increased likelihood of PCOS in subjects with higher serum concentrations of PFOA and PFOS (adjusted-ORs = 5.8–6.9, p < 0.05), and with lower urine concentrations of mBzP and mono-n-butyl phthalate (mBP) (aORs = 0.14–0.25, p < 0.05).

**Conclusions:**

Our data suggest that PCOS case-patients may differ from controls in their environmental contaminant profile. PCOS subjects had higher serum concentrations of two PFCs, PFOA and PFOS, and lower urine concentrations of mBP and mBzP. Future studies are needed to confirm these preliminary findings and determine if these chemicals or their precursors may have a role in the pathogenesis of PCOS.

**Electronic supplementary material:**

The online version of this article (doi:10.1186/1472-6823-14-86) contains supplementary material, which is available to authorized users.

## Background

Polycystic ovary syndrome (PCOS) is an endocrine-metabolic disorder that affects approximately 6-10% of women of child bearing age [1–4], approximately 4 million women in the United States, and is the most frequent cause of oligo-anovulatory infertility [5]. PCOS is characterized by ovulatory dysfunction, hirsutism, or hyperandrogenemia and is associated with insulin resistance, hyperinsulinemia, type 2 diabetes mellitus, and endometrial carcinoma [4, 6]. The annual cost of evaluating and providing care for PCOS to reproductive-aged women in the United States alone exceeds $4.3 billion [7].

Some pollutants may disrupt endocrine processes, but little is known about the effects of environmental contaminants on the development of PCOS. Organochlorine pesticides (OCPs) and bisphenol A (BPA) have been postulated as potential xenohormones in women by mimicking estrogen action and/or antagonizing testosterone action and potentially altering the secretions of follicle-stimulating hormone and luteinizing hormone [8]. I*n vitro* and animal studies have implicated polybrominated diphenyl ethers (PBDEs), phthalates, polychlorinated biphenyls (PCBs) and BPA as endocrine disruptors [9–14]. All of these industrial chemicals can be detected in food, water, or air, and diet is an important route of human exposure [15].

Considering the high prevalence of PCOS, the existence of an association between exposure to environmental chemicals and disease could have significant public health and economic impact. We investigated whether an increase in the likelihood of being diagnosed with PCOS may be associated with exposure to 36 PCBs, 9 OCPs, 11 PBDEs, 8 PFCs, 8 phthalates, and BPA as measured by concentrations in serum or urine.

## Methods

### Participants and setting

Between March 2007 and May 2008 the Center for Androgen-related Research and Discovery at Cedars-Sinai Medical Center (CSMC) in Los Angeles, CA recruited fifty-two women with PCOS and fifty controls through media advertisements and subspecialty clinics. Most (>90%) of PCOS patients agreed to store blood and urine samples for future studies. As part of the CSMC study, controls volunteered in response to advertising in the surrounding community and university for “healthy” women aged 25–45 years. All women were aged 18–45 years. Both PCOS patients and controls provided CSMC with information about their age, race, ethnicity, virilization and body mass index which are associated with case status. Participants enrolled in the CSMC study provided written informed consent to allow CSMC to provide Centers for Disease Control and Prevention (CDC) with anonymized data and samples for our nested pilot study. This study was approved by the Internal Review Board of the Centers for Disease Control and Prevention (CDC protocol #4918).

Exclusion criteria included concurrent pregnancy, the use of hormonal (including oral contraceptives) or other medications for the prior three months, diabetes, menopause, and inability to provide written consent.

We defined case-patients based on the strict National Institutes of Health 1990 criteria for PCOS, specifically including anovulation or oligo-ovulation; clinical (i.e., a modified Ferriman-Gallwey [mFG] hirsutism score >6) or laboratory evidence of hyperandrogenism; and the exclusion of related disorders (i.e. thyroid dysfunction, hyperprolactinemia, non-classic adrenal hyperplasia, androgen-secreting tumors, etc.), as previously described [16]. We defined controls as healthy women with long-term regular and predictable menstrual cycles, without hirsutism (i.e. mFG scores of ≤2), and without clinical and laboratory evidence of hyperandrogenism or other hormonal dysfunction.

### Measures, sample collection and handling

Recruited participants provided single spot urine and blood samples at CSMC. To avoid environmental contamination, participants were asked to open the urine containers when ready to provide a sample and to avoid touching the inside of the container. Serum samples where obtained by venipuncture performed using standard protocol and precautions, and the samples drawn into one 10 ml red top Vacutainer^®^ tube per participant. Tubes were placed upright and blood allowed to clot at room temperature for 60 min, after which they were centrifuged at 3,000 rpm (~1000 g) for 10 min. Using a disposable pipet, the serum was transferred to CDC-provided containers (10 mL amber glass bottle with Teflon-lined screw cap [Wheaton, Millville, NJ] Serum vial for serum PBDE content and 2 mL Nalgene^®^ [Rochester, NY] cryovials for serum PFCs and lipids content). The serum PBDE vials had been previously cleaned in a laboratory dish washer and baked at 300°C overnight and the screw caps had been rinsed with methanol [17]. After recapping, each container was frozen upright in a CDC-provided cardboard storage box at -70°C until shipping.

First morning void urine samples were collected in individually wrapped collection cups provided by the CDC. At the time of collection participants were asked to wash their hands with soap and water, to collect at least 10–15 ml of urine in the cup, to not remove the cap from the cup until ready to void, and to not touch the inside of the cup or cap. Using a sterile disposable plastic pipet, urine samples were transferred into prelabeled 5 ml Nalgene^®^ cryovials (for urinary Phthalates/BPA content) and 2 ml Nalgene^®^ cryovials (for urinary creatinine content). The urinary samples were then placed in CDC-provided storage boxes and frozen at -20°C until shipping.

All sample collection vials were labeled with preprinted bar-coded labels provided by the CDC, and the date and time of collection was added using a permanent marker. Operators used disposable powder-free nitrile gloves and surgical masks during sample collection and processing, and all collection and processing supplies were provided by the CDC to maximize the integrity of the samples. All sample processing was performed in a fume hood lined with aluminum foil to minimize contamination by ambient dust, as PBDEs are well known contaminants in indoor dust. The laboratory space was further thoroughly cleaned before any laboratory work was undertaken to minimize any dust or particulate matter that could contaminate the samples. All samples were shipped to the CDC on dry ice and upon arrival stored at -70°C until the time of analysis [17].

### Toxicologic analyses

Blood samples were analyzed for concentration of 36 PCBs, 9 OCPs, 11 PBDEs and 8 PFCs. Laboratory results of blood-agent concentration included both whole serum and lipid-adjusted concentration values of PCBs, OCPs and BDEs and serum only for PFCs.

Analytical determination of PCBs, OCPs and PBDEs in serum were performed by gas chromatography isotope dilution high resolution mass spectrometry, after solid phase extraction (SPE) and co-extracted lipid removal techniques [17]. Total cholesterol and triglycerides were measured using a Roche Hitachi Mod P Chemistry Analyzer (Roche Hitachi, Basel, Switzerland), using single-point, forced-through-zero calibration curves and Roche colorimetric methods as described in the product applications #11491458216 V15 (total cholesterol) and #04843673003 V13 (total triglycerides).

The measurements of PCBs, OCPs, and PBDEs were made in batches of twenty-four unknowns, three quality controls and three method blanks. The method blanks were used to track any analytical background during the sample preparation and the final analytical results were blank subtracted. The limits of detection (LODs) were calculated as standard deviations (SDs) of the method blanks analyzed in parallel with the unknowns after subtracting the average blank concentration or as the instrumental LOD in the absence of a detectable blank level (Additional file 1). The concentration ratio between 2,2′,4,4′,5-pentabromodipehnyl ether (BDE-99) and 2,2′,4,4′-tetrabromodipehnyl ether (BDE-47) can be used as an indicator of contamination during sample collection. The median ratio (BDE-99/BDE-47) in this study was 0.19 (range 0.10 – 0.36) which is consistent with a metabolized pattern and hence did not indicate contamination of indoor dust which is expected to have a ratio close to 1 (i.e. the concentration ratio between BDE-99 and BDE-47 in the commercial pentaBDE product used as a flame retardant).

We measured PFCs in serum using a modification of the on-line SPE coupled to high-performance liquid chromatography (HPLC)-isotope dilution tandem mass spectrometry (MS/MS) approach previously described in detail [18].

Urine samples were analyzed for 11 phthalate metabolites and for BPA (total concentrations) using a modification of the on-line SPE-high performance liquid chromatography-isotope dilution tandem mass spectrometry approaches described before [19, 20]. Urinary creatinine, used to adjust for the dilution of the urine, was measured using an enzymatic reaction on a Roche Hitachi 912 chemistry analyzer (Roche Hitachi, Basel, Switzerland).

### Statistical analyses

#### Epidemiologic analyses

Concentrations that were below the LOD were assigned a value equal to LOD/√2 [21]. No statistical analyses were conducted for compounds for which the majority (>50%) of samples had concentrations < LOD. We searched for PBDE, PCB, OCP, phthalate metabolites, PFCs and BPA concentrations that were significantly (p < 0.05) higher among PCOS case-patients compared to controls using Wilcoxon Rank-Sum.

### Multivariate logistic regression analyses

We used the serum concentrations or urine concentrations of the chemicals to categorize exposures to the chemicals or their parent compounds. We split observations into tertiles based on chemical concentrations then performed multivariable logistic regression analyses to explore associations between the concentrations of the chemicals (in μg/L for PFCs; lipid-adjusted for PCBs, OCPs and PBDEs and creatinine-adjusted for phthalate metabolites and BPA) and PCOS case status after adjusting for potential confounders (i.e. age [divided into 5 categories], body mass index [BMI], and White race [compared to all other races]). This multivariable logistic regression model is represented as PCOS (0/1) = α + β_1_(chemical concentration tertile) + β_2_(age) + β_3_(BMI) + β_4_(race). Hispanic ethnicity was not significantly related to PCOS when tested in preliminary analyses by logistic regression alone or when controlling for race, BMI, or age; therefore we did not statistically control for Hispanic ethnicity in the final models to conserve power. Due to the high ratio of variables in the model to study participants we calculated exact adjusted odds ratios, exact p values, and exact confidence intervals using the network method [22]. All statistical analyses were conducted using SAS v9.3 (Cary, NC). More than 50% of the samples had serum or urine concentrations < LOD for 22 PCBs, 3OCPs, 2 phthalate metabolites, and 2 PFCs; thus, no further analyses were conducted for these compounds (Additional file 2).

## Results

Comparing the 52 PCOS case-patients and 50 controls, we observed that women with PCOS had a greater mean BMI (OR = 1.1; 95% CI = 1.0–1.2) and were younger (OR = 0.7; 95% CI = 0.5–0.9) than controls (Table 1). Forty-four (85%) of 52 PCOS case-patients and 27 (54%) controls were white, 6 (12%) case-patients and 16 (32%) controls were Black, and two (4%) case-patients and six (12%) controls were Asian (p = 0.003). Thirty (58%) of 52 PCOS case-patients were Hispanic compared to 37 (74%) controls (p = 0.06). Case-patients and controls had similar total cholesterol (median 188 mg/dL [IQR 160–213]) and triglycerides (median 88 mg/dL [IQR 62–137]) (Table 2).

**Table 1:** **Minimum, maximum, mean and standard deviation values for body mass index (BMI, in Kg/m** ^**2**^ **) and age (in years) at time of recruitment for cases and controls**

		Mean (Range)	S.D.	t (control–case)	p-value ^a^
**BMI**	Cases	32.82 (18.9-51.2)	8.32	**-3.427**	0.001
	Controls	27.62 (17.5-47.1)	6.91		
**AGE**	Cases	28.12 (18.0-45.0)	6.04	**2.673**	0.009
	Controls	31.84 (20.0-45.0)	7.94		

^a^p-value comparing mean of cases to mean of controls.

**Table 2:** **Minimum, maximum, mean, median, and interquartile range values for serum whole weight, total lipid, cholesterol and triglycerides for cases and controls**

		Mean	Range	Median	Interquartile range
**Serum weight (g)**	Cases	1.97	1.86-2.04	1.99	1.94-2.00
	Controls	1.97	1.87-2.04	1.99	1.96-2.01
**Total lipid (mg/dL)**	Cases	607.09	404.9-1002.8	590.3	519.48-684.98
	Controls	589.82	347.8-830.2	560.4	491.05-691.53
**Cholesterol (mg/dL)**	Cases	1.89	129-287	186.5	165-213
	Controls	1.88	98-281	187.5	160-214
**Triglycerides (mg/dL)**	Cases	114.75	37-457	96.5	67.25-138.00
	Controls	99.8	24-223	77.0	58.25-135.25

Overall, more than 50% of participants had detectable serum or urine concentrations of 6 PBDEs, 14 PCBs, 6 OCPs, 11 phthalate metabolites, 4 PFCs, and BPA. Correlations between concentration values can be found in (Additional file 3).

### Perfluorinated compounds

PCOS case-patients had perfluorooctanoate (PFOA) and perfluorooctane sulfonate (PFOS) geometric mean serum-concentration higher than controls (4.1 μg/L vs. 2.3 μg/L, p = 0.001; and 8.2 μg/L vs. 4.9 μg/L, p = 0.01, respectively) (Table 3). Participants were 6.9-fold more likely to have PCOS if they had PFOA concentrations in the highest tertile (geometric mean = 5.5 μg/L, range = 4.1–13.4 μg/L) when compared to those in the lowest tertile (geometric mean = 1.6 μg/L, range = 0.2–2.6 μg/L). Participants were also 5.8-fold more likely to have PCOS if they had PFOS concentrations in the highest tertile (geometric mean = 12.5 μg/L, range = 8.6–27.9 μg/L) (Table 4).

**Table 3:** **Serum and lipid-adjusted concentrations of brominated diphenyl ethers, polychlorinated biphenyls, and persistent organic pesticides, and perfluorinated compounds in polycystic ovary syndrome (PCOS) case-patients and controls**

	Geometric mean whole weight concentration (pg/g serum)		Lipid-adjusted concentration (ng/g lipid)	
Compounds	Case-patients	Controls	Case-patients	Controls
**Brominated (Bi-) Diphenyl Ethers**				
2,4,4′-tribromodiphenyl ether (PBDE28)	10.8	9.9	1.9	1.8
2,2′,4,4′-tetrabromodiphenyl ether (PBDE47)	144.5	148.6	24.9	26.5
2,2′,4.4′,5-pentabromodiphenyl ether (PBDE99)	27.5	28.8	4.7	5.1
2,2′,4,4′,6-pentabromodiphenyl ether (PBDE100)	27.8	29.8	4.8	5.3
2,2′,4,4′,5,5′-hexabromobiphenyl (BB153)	3.3	6.0	0.6	1.0^a^
2,2′,4,4′,5,5′-hexabromodiphenyl (PBDE153)	29.7	34.3	5.1	6.1
**Polychlorinated Biphenyls**				
2,2′,4,4′,5-pentaCB (PCB99)	10.8	12.8	1.9	2.3
2,3,3′,4,4′-pentaCB (PCB105)	3.8	3.8	0.7	0.7
2,3′,4,4′,5-pentaCB (PCB118)	15.6	15.6	2.7	2.8
2,2′,3,4′,5,5′-hexaCB (PCB146)	4.2	5.0	0.7	0.9
2,2′,4,4′,5,5′-hexaCB (PCB153)	38.6	47.2	6.7	8.4
2,3,3′,4,4′,5-hexaCB (PCB156)	4.3	5.4	0.7	1.0
2,2′,3,4,4′,5′-hexaCB (PCB138-158)	31.5	35.7	5.4	6.4
2,2′,3,3′,4,4′,5-heptaCB (PCB170)	9.9	12.0	1.7	2.1
2,2′,3,4,4′,5,5′-heptaCB (PCB180)	25.1	29.7	4.3	5.3^b^
2,2′,3,4,4′,5′,6-heptaCB (PCB183)	3.8	4.4	0.7	0.8
2,2′,3,4′,5,5′,6-heptaCB (PCB187)	7.1	9.2	1.2	1.7
2,2′,3,3′,4,5,6,6′-octaCB (PCB199)	4.0	5.3	0.7	0.9
2,2′,3,3′,4,4′,5,5′-octaCB (PCB 194)	0.7	1.1	0.7	1.1
2,2′,3,3′,4,4′,5′,6-octaCB and 2,2′,3,4,4′,5,5′,6-octaCB (PCB196_203)	4.6	6.2	0.8	1.1
**Persistent pesticides**				
Hexachlorobenzene (HCB)	53.9	51.2	9.2	9.1
β-Hexachlorocyclohexane (B-HCCH)	16.0	16.1	4.2	4.4
Oxychlordane	18.6	23.1	3.2	4.1
Trans-Nonachlor	29.6	31.9	5.1	5.7
2,2-Bis(4-chlorophenyl)-1, 1-dichloroethene (PP DDE)	1217.5	1397.2	210.2	248.7
2,2-Bis(4-chlorophenyl-1, 1, 1-trichloroethane (PP DDT)	19.0	18.1	3.3	3.2
**Perfluorinated Compounds**	μg/L serum	μg/L serum		
Perfluorooctanoate (PFOA)	4.1	2.3^c^		
Perfluorooctane sulfonate (PFOS)	8.2	4.9^d^		
Perfluorohexane sulfonate (PFHxS)	1.1	0.7		
Perfluorononanoate (PFNA)	1.2	0.9		

^a^p = 0.02 where controls have higher BB153 levels than case-patients.

^b^p = 0.04 where controls have higher PCB180 levels than case-patients.

^c^p = 0.001 where case-patients have higher PFOA levels than controls.

^d^p = 0.01 where case-patients have higher PFOS levels than controls.

**Table 4:** **Logistic regression for middle and highest tertile serum concentrations compared to the lowest tertile for brominated diphenyl ethers, polychlorinated biphenyls, organochlorine pesticides, and perfluorinated compounds**

			Unadjusted concentrations									Lipid-adjusted concentrations					
Agent	N Case	N Control	Tertile	OR	95% C.I.	p	Adj OR ^a^	Exact 95% C.I	Exact p	N Case	N Control	OR	95% C.I.	p	Adj OR ^a^	Exact 95% C.I	Exact p
**Brominated Diphenyl Ethers**																	
2,4,4′-tribromodiphenyl ether (PBDE28)	15	19	Middle	0.79	0.30-2.05		0.54	0.14-1.90		19	16	1.32	0.51-3.41		1.04	0.29-3.68	
	20	14	Highest	1.43	0.55-3.72		0.95	0.27-3.31		15	18	1.00	0.38-2.68		0.69	0.17-2.64	
2,2′,4,4′-tetrabromodiphenyl ether (PBDE47)	17	17	Middle	1.00	0.39-2.59		0.67	0.18-2.30		16	18	0.79	0.30-2.05		0.65	0.17-2.38	
	18	16	Highest	1.12	0.43-2.91		0.70	0.21-2.76		18	16	1.00	0.39-2.59		0.73	0.20-2.60	
2,2′,4.4′,5-pentabromodiphenyl ether (PBDE99)	18	15	Middle	1.27	0.49-3.29		1.04	0.30-3.52		17	17	0.89	0.34-2.30		1.21	0.36-4.26	
	17	17	Highest	1.06	0.41-2.72		0.70	0.20-2.37		17	17	0.89	0.34-2.30		0.63	0.18-2.16	
2,2′,4,4′,6-pentabromodiphenyl ether (PBDE100)	18	16	Middle	1.00	0.39-2.59		0.68	0.18-2.38		20	14	1.43	0.55-3.72		0.90	0.25-3.14	
	16	18	Highest	0.79	0.31-2.05		0.65	0.17-2.40		15	19	0.79	0.30-2.05		0.66	0.17-2.50	
2,2′,4,4′,5,5′-hexabromobiphenyl ether (BB153)	23	13	Middle	1.24	0.47-3.25		1.26	0.35-4.60		17	12	0.85	0.32-2.26		1.10	0.30-4.14	
	9	23	Highest	**0.27**	0.10-0.77	0.01	0.31	0.04-2.15		10	23	**0.26**	0.10-0.69	0.01	0.49	0.08-2.90	
2,2′,4,4′,5,5′-hexabromodiphenyl ether (PBDE153)	19	16	Middle	1.06	0.41-2.72		1.29	0.39-4.45		17	17	0.70	0.27-1.83		1.03	0.30-3.65	
	15	18	Highest	0.74	0.28-1.94		1.11	0.31-4.04		15	19	0.55	0.21-1.44		1.01	0.27-3.94	
**Polychlorinated Biphenyls**																	
2,2′,4,4′,5-pentaCB (PCB99)	18	16	Middle	0.70	0.27-1.83		2.12	0.55-9.07		16	22	**0.31**	0.11-0.86	0.024	0.94	0.23-3.41	
	13	21	Highest	0.38	0.14-1.02	0.055	1.10	0.27-4.65		15	19	**0.34**	0.12-0.95	0.040	0.86	0.23-3.84	
2,3,3′,4,4′-pentaCB (PCB105)	19	15	Middle	1.00	0.38-2.60		1.19	0.34-4.26		16	16	0.86	0.34-2.19		0.92	0.27-3.14	
	14	20	Highest	0.55	0.21-1.45		0.79	0.22-2.83		15	16	0.80	0.31-2.07		1.16	0.34-4.09	
2,3′,4,4′,5-pentaCB (PCB118)	17	17	Middle	0.89	0.34-2.30		1.68	0.46-6.57		20	14	1.27	0.49-3.31		2.06	0.59-7.89	
	17	17	Highest	0.89	0.34-2.30		2.18	0.56-9.22		14	20	0.62	0.24-1.62		1.21	0.31-4.88	
2,2′,3,4′,5,5′-hexaCB (PCB146)	17	13	Middle	1.12	0.43-2.92		4.01	0.98-19.44	0.055	15	12	0.98	0.37-2.60		3.33	0.81-15.86	
	14	19	Highest	0.63	0.25-1.61		2.91	0.68-14.15		14	20	0.55	0.22-1.38		2.40	0.60-10.83	
2,2′,4,4′,5,5′-hexaCB (PCB153)	22	12	Middle	1.83	0.69-4.85		**5.79**	1.41-28.11	0.011	17	17	0.48	0.18-1.28		1.88	0.47-8.21	
	13	21	Highest	0.62	0.24-1.62		4.20	0.81-24.95		12	22	**0.26**	0.09-0.71	0.009	1.42	0.27-7.89	
2,3,3′,4,4′,5-hexaCB (PCB156)	21	14	Middle	1.33	0.51-3.46		3.40	0.91-14.21		20	14	1.05	0.40-2.78		2.25	0.62-8.79	
	13	20	Highest	0.58	0.22-1.52		4.50	0.76-31.05		13	22	0.44	0.16-1.15		2.58	0.45-16.22	
2,2′,3,4,4′,5′-hexaCB and 2,3,3′,4,4′,6-hexaCB (PCB138-158)	20	14	Middle	1.13	0.43-2.95		2.73	0.72-11.33		18	18	0.60	0.23-1.58		1.57	0.43-6.11	
	13	21	Highest	0.49	0.18-1.29		2.48	0.50-13.49		14	20	0.42	0.16-1.13		1.70	0.39-7.90	
2,2′,3,3′,4,4′,5-heptaCB (PCB170)	24	10	Middle	2.70	0.99-7.33	0.051	**6.88**	1.71-32.96	0.003	20	13	1.30	0.49-3.40		**5.13**	1.24-25.16	0.020
	12	22	Highest	0.61	0.23-1.62		4.04	0.64-29.31		13	21	0.52	0.20-1.36		4.97	0.78-37.25	
2,2′,3,4,4′,5,5′-heptaCB (PCB180)	11	23	Middle	2.35	0.88-6.30		**6.42**	1.60-30.52	0.005	19	14	1.02	0.39-2.66		**4.48**	1.08-21.73	0.037
	21	13	Highest	0.70	0.27-1.83		5.21	0.88-36.37		13	21	0.46	0.18-1.22		5.93	0.88-48.77	0.073
2,2′,3,4,4′,5′,6-heptaCB (PCB183)	22	12	Middle	1.83	0.69-4.85		**4.21**	1.11-18.26	0.017	21	12	1.65	0.63-4.36		3.49	0.94-14.53	0.065
	13	21	Highest	0.62	0.24-1.62		3.55	0.67-21.68		13	21	0.59	0.22-1.52		2.94	0.59-16.46	
2,2′,3,4′,5,5′,6-heptaCB (PCB187)	20	14	Middle	1.27	0.49-3.31		2.99	1.35-18.96		23	17	1.11	0.43-2.87		3.07	0.81-13.00	
	14	20	Highest	0.62	0.24-1.62		3.26	0.80-12.47		12	19	0.52	0.19-1.43		3.68	0.71-21.89	
2,2′,3,3′,4,4′,5,5′-octaCB (PCB194)	17	11	Middle	1.26	0.47-3.37		3.64	0.93-16.27	0.060	19	10	1.55	0.58-4.17		3.85	1.00-17.04	0.051
	13	21	Highest	0.51	0.20-1.29		3.45	0.61-22.50		11	22	0.41	0.16-1.06		2.86	0.48-19.26	
2,2′,3,3′,4,5,6,6′-octaCB (PCB199)	20	13	Middle	1.23	0.47-3.21		3.04	0.83-12.59		19	11	1.43	0.54-3.72		3.89	1.00-16.80	0.054
	12	21	Highest	0.46	0.17-1.20		2.04	0.43-10.71		11	21	0.43	0.16-1.12		2.31	0.48-12.61	
2,2′,3,3′,4,4′,5′,6-octaCB and 2,2′,3, 4, 4′,5,5′,6-octaCB (PCB196-203)	23	11	Middle	2.09	0.78-5.59		**7.50**	1.72-40.29	0.004	22	13	1.41	0.53-3.72		**4.19**	1.07-19.09	0.038
	12	22	Highest	0.54	0.21-1.44		4.67	0.78-33.23		12	22	0.45	0.17-1.21		3.28	0.56-21.38	
**Persistent Pesticides**																	
Hexachlorobenzene (HCB)	18	15	Middle	1.27	0.49-3.30		3.55	0.90-16.35		15	17	0.63	0.24-1.64		1.06	0.29-3.99	
	17	17	Highest	1.06	0.41-2.72		2.46	0.64-10.29		16	18	0.63	0.25-1.63		1.58	0.43-6.38	
β-Hexachlorocyclohexane (B-HCCH)	20	15	Middle	2.05	0.78-5.39		3.87	0.96-18.35		22	11	2.02	0.75-5.42		3.79	0.98-17.20	
	16	18	Highest	1.13	0.43-2.93		2.71	0.72-11.36		13	20	0.74	0.29-1.92		2.13	0.57-8.63	
Oxychlordane	18	16	Middle	0.89	0.34-2.31		2.16	0.59-8.60		19	14	1.00	0.37-2.65		3.21	0.81-14.64	
	15	19	Highest	0.62	0.24-1.62		2.61	0.55-14.25		14	20	0.52	0.20-1.36		2.21	0.47-11.56	
Trans-Nonachlor	18	16	Middle	1.00	0.39-2.59		2.19	0.60-8.67		18	16	0.89	0.34-2.31		2.17	0.58-8.80	
	16	18	Highest	0.79	0.30-2.05		3.16	0.64-18.15		15	19	0.62	0.24-1.62		1.95	0.43-9.67	
2,2-Bis(4-chlorophenyl)-1,	21	13	Middle	1.61	0.62-4.24		2.35	0.67-8.74		21	13	1.44	0.55-3.77		2.93	0.78-12.30	
1–dichloroethene (DDE)	14	20	Highest	0.70	0.27-1.82		1.15	0.31-4.37		13	21	0.55	0.21-1.44		1.14	0.30-4.44	
2,2-Bis(4-chlorophenyl-1, 1,	13	20	Middle	0.52	0.20-1.36		0.45	0.12-1.62		15	18	0.62	0.24-1.63		0.79	0.22-2.79	
1-trichloroethane (DDT)	19	14	Highest	1.09	0.42-2.82		1.29	0.35-4.84		17	16	0.80	0.31-2.07		1.02	0.30-3.52	
**Perfluorinated compounds**																	
PFOA	15	18	Middle	2.32	0.83-6.45		1.65	0.45-6.14									
Perfluorooctanoate	28	7	Highest	**11.11**	3.61-34.24	0.000	**6.93**	1.79-29.92	0.003								
PFOS	19	13	Middle	**3.80**	1.38-10.48	0.010	3.43	0.95-13.31	0.062								
Perfluorooctane sulfonate	23	11	Highest	**5.44**	1.95-15.13	0.001	**5.79**	1.58-24.12	0.005								
PFHxS	16	12	Middle	2.00	0.75-5.33		0.85	0.20-3.31									
Perfluorohexane sulfonate	20	14	Highest	2.14	0.84-5.44		1.20	0.35-4.07									
PFNA	17	13	Middle	2.15	0.80-5.73		1.13	0.37-4.49									
Perfluorononanoate	21	14	Highest	2.46	0.95-6.36		2.25	0.67-8.00									

^a^Controlling for age, BMI, and race.

Bold font highlights, for any individual agent, middle or highest tertile serum concentrations that are significantly different than the lowest tertile concentration.

### Polychlorinated biphenyls

Participants were 5.79-7.5 times more likely to have PCOS if they had PCB 153, 170, 180, 183, or 196 and 203 whole weight (pg/mL serum) concentrations in the middle tertile (but not the highest tertile), when compared to those in the lowest tertile (Table 4). A similar pattern also occurred among lipid-adjusted measurements of PCB 170, 180, and 196 and 203 concentrations. PCOS case-patients had PCB180 geometric mean lipid-adjusted concentrations (4.3 ng/g) *lower* than controls (5.3 ng/g) (p = 0.04) (Table 3).

### Phthalates

PCOS case-patients had monobenzyl phthalate (mBzP) geometric mean creatinine-adjusted urine concentrations (7.5 μg/g creatinine) *lower* than controls (11.7 μg/g creatinine) (p = 0.02) (Table 5). After controlling for BMI, age, and race, participants were *less* likely to have PCOS if they had creatinine-adjusted urine concentrations of mBzP or mono-n-butyl phthalate (mBP) in the middle or highest tertile compared to the lowest and mono-2-ethylhexyl phthalate (mEHP), and monoethyl phthalate (mEP) in the middle (but not the highest) tertile compared to the lowest (Table 6).

**Table 5:** **Urinary concentrations of phthalate metabolites and bisphenol A in polycystic ovary syndrome (PCOS) case-patients and controls**

Agents		Geometric mean concentration (μg/L)		Creatinine-adjusted geometric mean concentration (μg/g creatinine)	
Phthalates	Phthalate metabolites measured	PCOS case-patients	PCOS control-patients	PCOS case-patients	PCOS control-patients
Butylbenzyl phthalate (BBzP)	Monobenzyl phthalate (mBzP)	4.7	9.0	7.5	11.7^a^
Di-n-butyl phthalate (DBP)	Mono-n-butyl phthalate (mBP)	15.3	24.8	17.7	23.2
Diethyl phthalate (DEP)	Monoethyl phthalate (mEP)	103.7	138.3	181.1	195.8
Di-isodecyl phthalate (DiDP)	Mono(carboxynonyl) phthalate (mCNP)	3.2	3.0	3.6	2.6
Di-isononyl phthalate (DiNP)	Mono(carboxyoctyl) phthalate (mCOP)	6.7	9.0	7.8	8.4
Di-n-octyl phthalate (DOP)	Mono-3-carboxypropyl phthalate (mCPP)	2.4	3.6	2.8	3.4
Di-2-ethylhexyl phthalate (DEHP)	Mono-2-ethyl-5-carboxypentyl phthalate (mECPP)	41.5	43.2	47.9	40.4
	Mono-2-ethyl-5-hydroxyhexyl phthalate (mEHHP)	23.7	28.3	27.3	26.4
	Mono-2-ethylhexyl phthalate (mEHP)	3.3	4.0	3.2	3.5
	Mono-2-ethyl-5-oxohexyl phthalate (mEOHP)	14.0	17.4	16.2	16.3
Di-isobutyl phthalate (DiBP)	Mono-isobutyl phthalate (miBP)	6.0	8.7	7.0	8.2
**Phenols**					
bisphenol A (BPA)		1.6	2.1	1.6	1.9

^a^p = 0.02 where controls have higher mBzP urinary concentrations than case-patients.

**Table 6:** **Logistic regression for middle and highest tertile creatinine-adjusted urine concentrations compared to the lowest tertile for phthalate metabolites and bisphenol A**

Agent	N Cases	N Controls	Tertile	Odds ratio	95% C.I.		Adjusted OR ^a^	Exact 95% C.I	Exact p-value
**Phthalate metabolites**									
mBzP	16	18	Middle	0.43	0.16-1.14		**0.24**	0.06-0.86	0.025
Monobenzyl phthalate	13	20	Highest	**0.27**	0.10-0.75	0.012	**0.15**	0.03-0.58	0.004
mBP	15	19	Middle	**0.21**	0.07-0.58	0.012	**0.14**	0.03-0.54	0.002
Mono-n-butyl phthalate	14	19	Highest	**0.41**	0.15-1.12	0.003	**0.25**	0.06-0.96	0.042
mEHP	11	20	Middle	**0.22**	0.08-0.65	0.006	**0.17**	0.04-0.63	0.005
Mono-2-ethylhexyl phthalate	16	17	Highest	0.87	0.34-2.21		0.91	0.26-3.19	
mEP	16	18	Middle	**0.27**	0.10-0.75	0.012	**0.12**	0.02-0.48	0.001
Monoethyl phthalate	15	18	Highest	0.66	0.25-1.75		0.30	0.06-1.18	
mCNP	16	19	Middle	1.00	0.39-2.59		0.80	0.23-2.71	
Mono (carboxynonyl) phthalate	18	14	Highest	1.06	0.41-2.77		1.45	0.43-5.07	
mCOP	18	16	Middle	**2.82**	1.05-7.60	0.040	2.27	0.61-8.84	
Mono (carboxyoctyl) phthalate	14	18	Highest	1.45	0.55-3.85		1.58	0.46-5.55	
mCPP	16	14	Middle	0.78	0.30-2.07		0.83	0.23-2.91	
Mono-3-carboxypropyl phthalate	14	19	Highest	0.54	0.21-1.44		0.36	0.09-1.28	
mECPP	16	17	Middle	0.95	0.36-2.45		0.78	0.22-2.73	
Mono-2-ethyl-5-carboxypentyl	16	17	Highest	1.85	0.69-4.91		2.09	0.57-8.16	
mEHHP	15	19	Middle	0.66	0.25-1.72		0.50	0.15-1.66	
Mono-2-ethyl-5-hydroxyhexyl	16	17	Highest	1.00	0.38-2.63		1.27	0.34-4.96	
mEOHP	16	17	Middle	0.94	0.36-2.45		0.88	0.27-2.87	
Mono-2-ethyl-5-oxohexyl phthalate	15	18	Highest	0.84	0.32-2.18		1.07	0.29-4.00	
miBP	17	17	Middle	0.58	0.22-1.54		0.56	0.16-1.91	
Mono-isobutyl phthalate	12	21	Highest	0.40	0.15-1.08		0.31	0.08-1.08	
**Phenols**									
BPA	15	16	Middle	0.43	0.16-1.15		0.44	0.13-1.46	
Bisphenol A	14	20	Highest	0.84	0.32-2.21		0.73	0.20-2.58	

^a^Controlling for age, BMI, and race.

Bold font highlights, for any individual agent, middle or highest tertile serum concentrations that are significantly different than the lowest tertile concentration.

### Other findings

PCOS case-patients and controls had similar geometric mean lipid-adjusted PBDEs, and OCPs serum concentrations (Table 3), and urinary BPA concentrations (Table 5), with the exception of BB153 of which controls had higher concentrations than case-patients (Table 3). After controlling for BMI, age, and ethnicity, no increase in likelihood for PCOS was associated with any of the measured PBDEs, OCPs, or BPA, with one exception (Tables 4 &6).

## Discussion

### Key results

Our findings suggest that increasing odds of PCOS case-status was associated with higher serum concentrations of two PFCs (PFOA and PFOS) and with several PCB congeners. PFOS, PFOA, PCBs and phthalates have all been implicated as endocrine disruptors and have been linked to other reproductive outcomes. Previous epidemiologic investigations suggest that exposure to PFCs may increase women’s risk of early menopause [23], thyroid disease [24], and delayed pregnancy and subfecundity [25–27]. Exposure to certain PCBs has been associated with other reproductive health effects such as delayed pregnancy [28]. PCB exposure has also been linked to increased breast cancer [29], diabetes and thyroid disease [30] risk. This is the first documented evidence of an association between PCOS and serum concentrations of PFCs or PCBs.

PCOS case-patients had urinary mBzP concentration lower than controls, and *lower* concentrations of mBzP, mBP, mEHP and mEP were associated with an increased likelihood of PCOS. Previous research suggests that some phthalates, including di-2-ethylhexyl phthalate (DEHP), the precursor of mEHP, have anti-androgenic effects in animals [12]. Also, epidemiologic studies have linked mEHP and a metabolite of di-isononyl phthalate (DiNP) with decreased testosterone production in men [31] and mBP, mono-isobutyl phthalate, mBzP, and the sum of metabolites of DEHP and of DiNP with delayed pubarche in women [32]. PCOS is characterized by hyperandrogenemia; therefore, our results are consistent with previous evidence of the anti-androgenic effects of certain phthalates.

Unlike previous studies [33–35], we found no association between PCOS and BPA. Previous investigators quantified serum BPA concentrations using a commercially available enzyme-linked immunosorbent assay (ELISA) kit, whereas we measured urinary concentrations of this toxicant using the gold-standard detection technique, isotope dilution mass spectrometry [20]. ELISA lacks adequate analytical selectivity and specificity, and because matrix effects may induce performance anomalies, ELISA is not adequate for the quantitative determination of BPA in clinical specimens [34, 36].

### Limitations

This study is subject to several limitations. First, our sample size of 52 PCOS case-patients and 50 controls may not be large enough to generate enough statistical power to detect a difference in some of the toxicant concentrations among PCOS case-patients and controls. Similarly, the small sample size limits the possibility of considering non-monotonic dose–response relationships and some significant associations can be chance findings due to the large number of statistical tests.

We obtained a single spot serum and urine specimen. Therefore, the concentrations of each toxicant represent a snapshot of each woman’s exposure at a given time. A single measurement design may be ineffective in detecting an association between PCOS and pollutants that metabolize quickly. For example, some of the measured analytes (such as PCBs, PBDEs, OCPs, and PFCs) persist in the body for years, while phthalates and BPA metabolize quickly and are eliminated from urine within a few hours after exposure.

It is unclear whether the observed associations suggest that PFOA, PFOS, and the implicated PCB congeners increase risk for PCOS or whether the endocrine milieu of the disorder alters the storage and clearance of these chemicals, leading to increased serum measurements in PCOS patients. Oligomenorrhea and amenorrhea are common symptoms of PCOS. Thus, PCOS cases, who menstruate less frequently compared to controls, may have similar exposures to women without PCOS, but higher blood-toxicant concentrations [23]. PCBs, however, are stored mostly in adipose tissue making amenorrhea an unlikely explanation for differences in measured exposure.

Finally, a larger proportion of case-patients were of White race compared to controls, and PCOS patients were younger and had significantly higher BMI than controls, although we attempted to control for these parameters known to be associated with case-status in the logistic regression models.

## Conclusions

In summary, associations between PCOS and serum concentrations of PFOS and PFOA as well as some PCBs were observed in this pilot study. This research highlights the need to further substantiate the association between PCOS and exposure to these pollutants using different study designs such as including large cohorts and measuring for additional confounders such as adiposity. The relationships between environmental contaminants are complex. Humans are exposed to many endocrine disrupting chemicals at once. Some chemicals have androgenic effects while others have anti-androgenic effects and for some chemicals those effects are indirect, rather than being a direct agonist or antagonist against specific hormone receptors. It is, therefore, difficult to evaluate true associations between environmental contaminants and disease. Thus, further studies are needed to explore the potential mechanisms by which these chemicals might contribute to the development of PCOS.

## Electronic supplementary material

Additional file 1: **Limits of Detection (LODs).** (DOCX 21 KB)

Additional file 2: **Fifty percent or more of the samples examined had concentrations below the limit of detection (LOD) for these chemicals.** (DOCX 17 KB)

Additional file 3: **Correlations of brominated diphenyl ethers (BDE), polychlorinated biphenyls (PCB), organochlorine pesticides (OCP), perfluorinated compounds (PFC), phthalates, and bisphenol A.** (XLS 88 KB)

## References

[1] Asuncion M, Calvo RM, San Millan JL, Sancho J, Avila S, Escobar-Morreale HF (2000). A prospective study of the prevalence of the polycystic ovary syndrome in unselected Caucasian women from Spain. J Clin Endocrinol Metab.

[2] Azziz R, Woods KS, Reyna R, Key TJ, Knochenhauer ES, Yildiz BO (2004). The prevalence and features of the polycystic ovary syndrome in an unselected population. J Clin Endocrinol Metab.

[3] Diamanti-Kandarakis E, Kouli CR, Bergiele AT, Filandra FA, Tsianateli TC, Spina GG, Zapanti ED, Bartzis MI (1999). A survey of the polycystic ovary syndrome in the Greek island of Lesbos: hormonal and metabolic profile. J Clin Endocrinol Metab.

[4] Knochenhauer ES, Key TJ, Kahsar-Miller M, Waggoner W, Boots LR, Azziz R (1998). Prevalence of the polycystic ovary syndrome in unselected black and white women of the southeastern United States: a prospective study. J Clin Endocrinol Metab.

[5] Hull MG (1987). Epidemiology of infertility and polycystic ovarian disease: endocrinological and demographic studies. Gynecol Endocrinol.

[6] Wild RA (2002). Long-term health consequences of PCOS. Hum Reprod Update.

[7] Azziz R, Marin C, Hoq L, Badamgarav E, Song P (2005). Health care-related economic burden of the polycystic ovary syndrome during the reproductive life span. J Clin Endocrinol Metab.

[8] Massaad C, Entezami F, Massade L, Benahmed M, Olivennes F, Barouki R, Hamamah S (2002). How can chemical compounds alter human fertility?. Eur J Obstet Gynecol Reprod Biol.

[9] Shelby MD (2008). NTP-CERHR monograph on the potential human reproductive and developmental effects of bisphenol A. Ntp Cerhr Mon.

[10] Kodavanti PR (2005). Neurotoxicity of persistent organic pollutants: possible mode(s) of action and further considerations. Dose Response.

[11] Hunt PA, Lawson C, Gieske M, Murdoch B, Smith H, Marre A, Hassold T, VandeVoort CA (2012). Bisphenol A alters early oogenesis and follicle formation in the fetal ovary of the rhesus monkey. Proc Natl Acad Sci U S A.

[12] Parks LG, Ostby JS, Lambright CR, Abbott BD, Klinefelter GR, Barlow NJ, Gray LE (2000). The plasticizer diethylhexyl phthalate induces malformations by decreasing fetal testosterone synthesis during sexual differentiation in the male rat. Toxicol Sci.

[13] Casals-Casas C, Desvergne B (2011). Endocrine disruptors: from endocrine to metabolic disruption. Annu Rev Physiol.

[14] Hines EP, White SS, Stanko JP, Gibbs-Flournoy EA, Lau C, Fenton SE (2009). Phenotypic dichotomy following developmental exposure to perfluorooctanoic acid (PFOA) in female CD-1 mice: Low doses induce elevated serum leptin and insulin, and overweight in mid-life. Mol Cell Endocrinol.

[15] Prevention CfDCa, CDC (2012). Fourth National Report on Human Exposure to Environmental Chemicals, Updated Tables.

[16] Azziz R, Sanchez LA, Knochenhauer ES, Moran C, Lazenby J, Stephens KC, Taylor K, Boots LR (2004). Androgen excess in women: experience with over 1000 consecutive patients. J Clin Endocrinol Metab.

[17] Sjodin A, Jones RS, Lapeza CR, Focant JF, McGahee EE, Patterson DG (2004). Semiautomated high-throughput extraction and cleanup method for the measurement of polybrominated diphenyl ethers, polybrominated biphenyls, and polychlorinated biphenyls in human serum. Anal Chem.

[18] Kuklenyik Z, Needham LL, Calafat AM (2005). Measurement of 18 perfluorinated organic acids and amides in human serum using on-line solid-phase extraction. Anal Chem.

[19] Silva MJ, Samandar E, Preau JL, Reidy JA, Needham LL, Calafat AM (2007). Quantification of 22 phthalate metabolites in human urine. J Chromatogr B Analyt Technol Biomed Life Sci.

[20] Ye X, Kuklenyik Z, Needham LL, Calafat AM (2005). Automated on-line column-switching HPLC-MS/MS method with peak focusing for the determination of nine environmental phenols in urine. Anal Chem.

[21] Hornung RW RL (1990). Estimation of average concentration in the presence of nondetectable values. Appl Occup Environ Hyg.

[22] Mehta CR, Patel NR (1995). Exact logistic regression: theory and examples. Stat Med.

[23] Knox SS, Jackson T, Javins B, Frisbee SJ, Shankar A, Ducatman AM (2011). Implications of early menopause in women exposed to perfluorocarbons. J Clin Endocrinol Metab.

[24] Melzer D, Rice N, Depledge MH, Henley WE, Galloway TS (2010). Association between serum perfluorooctanoic acid (PFOA) and thyroid disease in the U.S. National Health and Nutrition Examination Survey. Environ Health Perspect.

[25] Fei C, McLaughlin JK, Lipworth L, Olsen J (2009). Maternal levels of perfluorinated chemicals and subfecundity. Hum Reprod.

[26] Vestergaard S, Nielsen F, Andersson AM, Hjollund NH, Grandjean P, Andersen HR, Jensen TK (2012). Association between perfluorinated compounds and time to pregnancy in a prospective cohort of Danish couples attempting to conceive. Hum Reprod.

[27] Whitworth KW, Haug LS, Baird DD, Becher G, Hoppin JA, Skjaerven R, Thomsen C, Eggesbo M, Travlos G, Wilson R, Longnecker MP (2012). Perfluorinated compounds and subfecundity in pregnant women. Epidemiology.

[28] Cohn BA, Cirillo PM, Sholtz RI, Ferrara A, Park JS, Schwingl PJ (2011). Polychlorinated biphenyl (PCB) exposure in mothers and time to pregnancy in daughters. Reprod Toxicol.

[29] Recio-Vega R, Velazco-Rodriguez V, Ocampo-Gomez G, Hernandez-Gonzalez S, Ruiz-Flores P, Lopez-Marquez F (2011). Serum levels of polychlorinated biphenyls in Mexican women and breast cancer risk. J Appl Toxicol.

[30] Donato F, Zani C (2010). [Chronic exposure to organochlorine compounds and health effects in adults: diabetes and thyroid diseases]. Ann Ig.

[31] Joensen UN, Frederiksen H, Jensen MB, Lauritsen MP, Olesen IA, Lassen TH, Andersson AM, Jorgensen N (2012). Phthalate excretion pattern and testicular function: a study of 881 healthy Danish men. Environ Health Perspect.

[32] Frederiksen H, Sorensen K, Mouritsen A, Aksglaede L, Hagen CP, Petersen JH, Skakkebaek NE, Andersson AM, Juul A (2012). High urinary phthalate concentration associated with delayed pubarche in girls. Int J Androl.

[33] Kandaraki E, Chatzigeorgiou A, Livadas S, Palioura E, Economou F, Koutsilieris M, Palimeri S, Panidis D, Diamanti-Kandarakis E (2011). Endocrine disruptors and polycystic ovary syndrome (PCOS): elevated serum levels of bisphenol A in women with PCOS. J Clin Endocrinol Metab.

[34] Tsutsumi O (2005). Assessment of human contamination of estrogenic endocrine-disrupting chemicals and their risk for human reproduction. J Steroid Biochem Mol Biol.

[35] Takeuchi T, Tsutsumi O, Ikezuki Y, Takai Y, Taketani Y (2004). Positive relationship between androgen and the endocrine disruptor, bisphenol A, in normal women and women with ovarian dysfunction. Endocr J.

[36] WHO (World Health Organization) (2010). Joint FAO/WHO Expert Meeting to Review Toxicological and Health Aspects of Bisphenol A: Summary Report including Report of Stakeholders Meeting on Bisphenol A.

[CR37] The pre-publication history for this paper can be accessed here: http://www.biomedcentral.com/1472-6823/14/86/prepub

