# Supplementary material for: Exploring the potential association between brominated diphenyl ethers, polychlorinated biphenyls, organochlorine pesticides, perfluorinated compounds, phthalates, and bisphenol a in polycystic ovary syndrome: a case–control study
Source: BMC Endocr Disord. 2014 Oct 28;14:86. doi: 10.1186/1472-6823-14-86 (PMC4287339; doi:10.1186/1472-6823-14-86)
Supplement: Supplementary file 1 — Additional file 1: Limits of Detection (LODs). (DOCX 21 KB) [file 12902_2014_298_MOESM1_ESM.docx]

**Limits of Detection (LODs):**

|  | |  | **Serum** | | | | **Lipid-adjusted** | | |
| --- | --- | --- | --- | --- | --- | --- | --- | --- | --- |
| **Abbreviation** | **Analyte name** | | **LOD (pg/mL serum)** | | | | **LOD (ng/g lipid)** | | |
| **Brominated (Bi-) Diphenyl Ethers** | | |  | | | |  | | |
| PBDE17 | 2,2',4- tribromodiphenyl ether | | 2.5-2.7 | | | | 0.3-0.8 | | |
| PBDE28 | 2,4,4'-tribromodiphenyl ether | | 2.5-2.7 | | | | 0.3-0.8 | | |
| PBDE47 | 2,2',4,4'-tetrabromodiphenyl ether | | 19.0-20.8 | | | | 2.0-6.1 | | |
| PBDE66 | 2,3',4',4-tetrabromodiphenyl ether | | 2.5-2.7 | | | | 0.3-0.8 | | |
| PBDE85 | 2.2',3,4,4'-pentabromodiphenyl ether | | 2.5-2.7 | | | | 0.3-0.8 | | |
| PBDE99 | 2,2',4.4',5-pentabromodiphenyl ether | | 16.2-17.8 | | | | 1.7-5.2 | | |
| PBDE100 | 2,2',4,4',6-pentabromodiphenyl ether | | 2.5-2.7 | | | | 0.3-0.8 | | |
| BB153 | 2,2',4,4',5,5'-hexabromobipheny ether | | 2.5-2.7 | | | | 0.3-0.8 | | |
| PBDE153 | 2,2',4,4',5,5'-hexabromodiphenyl ether | | 4.3-4.7 | | | | 0.5-1.4 | | |
| PBDE154 | 2,2',4,4',5,6'-hexabromodiphenyl ether | | 2.5-2.7 | | | | 0.3-0.8 | | |
| PBDE183 | 2,2',3,4,4',5',6-heptabromodiphenyl ether | | 2.5-2.7 | | | | 0.3-0.8 | | |
| **Polychlorinated Biphenyls** | | |  | | | |  | | |
| PCB18 | 2,2',5-triCB | | --- | | | | --- | | |
| PCB28 | 2,4,4'-triCB | | 46-50 | | | | 4.8-14.5 | | |
| PCB44 | 2,2',3,5'-tetraCB | | 8.6-9.5 | | | | 0.9-2.7 | | |
| PCB49 | 2,2',4,5'-tetraCB | | 6.6-7.3 | | | | 0.7-2.1 | | |
| PCB52 | 2,2',5,5'-tetraCB | | 13.8-15.1 | | | | 1.4-4.4 | | |
| PCB66 | 2,3',4,4'-tetraCB | | 18.5-20.3 | | | | 1.9-5.9 | | |
| PCB74 | 2,4,4',5-tetraCB | | 13.4-14.7 | | | | 1.4-4.3 | | |
| PCB87 | 2,2',3,4,5'-pentaCB | | 3.4-3.8 | | | | 0.4-1.1 | | |
| PCB99 | 2,2',4,4',5-pentaCB | | 2.5-2.7 | | | | 0.3-0.8 | | |
| PCB101 | 2,2',4,5,5'-pentaCB | | 3.8-4.1 | | | | 0.4-1.2 | | |
| PCB105 | 2,3,3',4,4'-pentaCB | | 2.5-2.7 | | | | 0.3-0.8 | | |
| PCB110 | 2,3,3',4',6-pentaCB | | 3.3-3.6 | | | | 0.3-1.1 | | |
| PCB118 | 2,3',4,4',5-pentaCB | | 4.5-4.9 | | | | 0.5-1.4 | | |
| PCB128 | 2,2',3,3',4,4'-hexaCB | | 2.5-2.7 | | | | 0.3-0.8 | | |
| PCB146 | 2,2',3,4',5,5'-hexaCB | | 2.5-2.7 | | | | 0.3-0.8 | | |
| PCB149 | 2,2',3,4',5',6-hexaCB | | 2.5-2.7 | | | | 0.3-0.8 | | |
| PCB151 | 2,2',3,5,5',6-hexaCB | | 2.5-2.7 | | | | 0.3-0.8 | | |
| PCB153 | 2,2',4,4',5,5'-hexaCB | | 2.5-2.7 | | | | 0.3-0.8 | | |
| PCB156 | 2,3,3',4,4',5-hexaCB | | 2.5-2.7 | | | | 0.3-0.8 | | |
| PCB157 | 2,3,3',4,4',5'-hexaCB | | 2.5-2.7 | | | | 0.3-0.8 | | |
| PCB138-158 | 2,2',3,4,4',5'-hexaCB | | 2.5-2.7 | | | | 0.3-0.8 | | |
| PCB167 | 2,3',4,4',5,5'-hexaCB | | 2.5-2.7 | | | | 0.3-0.8 | | |
| PCB170 | 2,2',3,3',4,4',5-heptaCB | | 2.5-2.7 | | | | 0.3-0.8 | | |
| PCB172 | 2,2',3,3',4,5,5'-heptaCB | | 2.5-2.7 | | | | 0.3-0.8 | | |
| PCB177 | 2,2',3,3',4',5,6-heptaCB | | 2.5-2.7 | | | | 0.3-0.8 | | |
| PCB178 | 2,2',3,3',5,5',6-heptaCB | | 2.5-2.7 | | | | 0.3-0.8 | | |
| PCB180 | 2,2',3,4,4',5,5'-heptaCB | | 2.5-2.7 | | | | 0.3-0.8 | | |
| PCB183 | 2,2',3,4,4',5',6-heptaCB | | 2.5-2.7 | | | | 0.3-0.8 | | |
| PCB187 | 2,2',3,4',5,5',6-heptaCB | | 2.5-2.7 | | | | 0.3-0.8 | | |
| PCB189 | 2,3,3’,4,4’,5,5’-heptaCB | | 2.5-2.7 | | | | 0.3-0.8 | | |
| PCB194 | 2,2',3,3',4,4',5,5'-octaCB | | 2.5-2.7 | | | | 0.3-0.8 | | |
| PCB195 | 2,2',3,3',4,4',5,6-octaCB | | 2.5-2.7 | | | | 0.3-0.8 | | |
| PCB196-203 | 2,2',3,3',4,4',5',6-octaCB and 2,2',3,4,4',5,5',6-octaCB | | 2.5-2.7 | | | | 0.3-0.8 | | |
| PCB199 | 2,2',3,3',4,5,6,6'-octaCB | | 2.5-2.7 | | | | 0.3-0.8 | | |
| PCB206 | 2,2',3.3',4,4',5,5',6-nonaCB | | 2.5-2.7 | | | | 0.3-0.8 | | |
| PCB209 | decaCB | | 2.5-2.7 | | | | 0.3-0.8 | | |
| **Persistent pesticides** |  | |  | | | |  | | |
| HCB | Hexachlorobenzene | | 13.1-14.3 | | | | 1.4-4.2 | | |
| B-HCCH | β-Hexachlorocyclohexane | | 8.4-9.2 | | | | 1.3-3.9 | | |
| G-HCCH | γ-Hexachlorocyclohexane (Lindane) | | 12.3-13.5 | | | | 1.3-3.9 | | |
| Oxychlor | Oxychlordane | | 12.3-13.5 | | | | 1.3-3.9 | | |
| T-Nona | Trans-Nonachlor | | 12.3-13.5 | | | | 1.3-3.9 | | |
| PP_DDE | 2,2-Bis(4-chloropheny1)-I, I -dichloroethene | | 4.9-5.4 | | | | 1.0-2.0 | | |
| PP_DDT | 2,2-Bis(4-chlorophenyl-I , I, I-trichloroethan | | 12.3-13.5 | | | | 1.3-3.9 | | |
| OP-DDT | 2-(4-chlorophenyl)-2-(2-chlorophenyl)- 1, 1, 1-trichloroethan | | 12.3-13.5 | | | | 1.3-3.9 | | |
| MIREX | Mirex | | 12.3-13.5 | | | | 1.3-3.9 | | |
|  |  | |  | | | |  | | |
|  |  | |  | | | |  | | |
| Perfluorinated compounds (in μg/L) | | |  | | | |  |  |  |
| PFOA | perfluorooctanoate acid | | 0.1 | | | |  |  |  |
| PFOS | perfluorooctane sulfonate | | 0.2 | | | |  |  |  |
| Et-PFOSA-AcOH | 2-(N-ethyl-perfluorooctane sulfonamido) acetate | | 0.2 | | | |  |  |  |
| Me-PFOSA-AcOH | 2-(N-methyl-perfluorooctane sulfonamido) acetate | | 0.2 | | | |  |  |  |
| PFDeA | Perfluorodecanoate | | 0.2 | | | |  |  |  |
| PFHxS | perfluorooctane sulfonamide | | 0.1 | | | |  |  |  |
| PFNA | Perfluorononanoate | | 0.1 | | | |  |  |  |
| PFOSA | perfluorooctane sulfonamide | | 0.1 | | | |  |  |  |
|  | |  | | **Urinary** | |  | | |  |
| **Abbreviation** | **Analyte name** | | | **LOD (μg/L)** | |  | | |  |
| **Phthalate metabolites** |  | | |  | | |  | | |
| mBzP | Monobenzyl phthalate | | | 0.3 | |  |  |  |  |
| mBP | Mono-n-butyl phthalate | | | 0.6 | |  |  |  |  |
| mEHP | Mono-2-ethylhexyl phthalate | | | 1.2 | |  |  |  |  |
| mEP | Monoethyl phthalate | | | 0.8 | |  |  |  |  |
| mECPP | Mono-2-ethyl-5-carboxypentyl phthalate | | | 0.6 | |  |  |  |  |
| mEHHP | Mono-2-ethyl-5-hydroxyhexyl phthalate | | | 0.7 | |  |  |  |  |
| mEOHP | Mono-2-ethyl-5-oxohexyl phthalate | | | 0.7 | |  |  |  |  |
| mCPP | Mono-3-carboxypropyl phthalate | | | 0.2 | |  |  |  |  |
| miBP | Mono-isobutyl phthalate | | | 0.3 | |  |  |  |  |
| mCOP | Monocarboxyoctyl phthalate | | | 0.7 | |  |  |  |  |
| mCNP | Monocarboxynonyl phthalate | | | 0.6 | |  |  |  |  |
| **Phenols** |  | | |  | | |  | | |
| BPA | Bisphenol A | | | 0.4 | |  |  |  |  |
|  | | | | |  | | | |  |
| * Results presented as a range where LODs are determined by blanks and sample size | | | | |  | | | |  |
