# Supplementary material for: Exploring the potential association between brominated diphenyl ethers, polychlorinated biphenyls, organochlorine pesticides, perfluorinated compounds, phthalates, and bisphenol a in polycystic ovary syndrome: a case–control study
Source: BMC Endocr Disord. 2014 Oct 28;14:86. doi: 10.1186/1472-6823-14-86 (PMC4287339; doi:10.1186/1472-6823-14-86)
Supplement: Supplementary file 2 — Additional file 2: Fifty percent or more of the samples examined had concentrations below the limit of detection (LOD) for these chemicals. (DOCX 17 KB) [file 12902_2014_298_MOESM2_ESM.docx]

**Fifty percent or more of the samples examined had concentrations below the limit of detection (LOD) for these chemicals.**

| **Agent** | **Agent Name** | **Percent detectable** | **Min** | **Serum Max** | **Lipid Max** |
| --- | --- | --- | --- | --- | --- |
| **Brominated Diphenyl Ethers** | |  |  |  |  |
| PBDE17 | 2,2',4- tribromodiphenyl ether | 8.8% | <LOD | 23.0 | 4.0 |
| PBDE66 | 2,3',4',4-tetrabromodiphenyl ether | 28.4% | <LOD | 14.0 | 2.3 |
| PBDE85 | 2.2',3,4,4'-pentabromodiphenyl ether | 47.1% | <LOD | 67.6 | 11.6 |
| PBDE154 | 2,2',4,4',5,6'-hexabromodiphenyl ether | 49.0% | <LOD | 51.3 | 8.8 |
| PBDE183 | 2,2',3,4,4',5',6-heptabromodiphenyl ether | 12.7% | <LOD | 9.3 | 1.6 |
| **Polychlorinated Biphenyls** | |  |  |  |  |
| PCB18 | 2,2',5-triCB | 0.0% | NR | NR | NR |
| PCB28 | 2,4,4'-triCB | 2.0% | <LOD | 59.1 | 15.0 |
| PCB44 | 2,2',3,5'-tetraCB | 0.0% | <LOD | <LOD | <LOD |
| PCB49 | 2,2',4,5'-tetraCB | 0.0% | <LOD | <LOD | <LOD |
| PCB52 | 2,2',5,5'-tetraCB | 0.0% | <LOD | <LOD | <LOD |
| PCB66 | 2,3',4,4'-tetraCB | 0.0% | <LOD | <LOD | <LOD |
| PCB74 | 2,4,4',5-tetraCB | 38.2% | <LOD | 164.8 | 26.1 |
| PCB87 | 2,2',3,4,5'-pentaCB | 3.9% | <LOD | 6.6 | 1.5 |
| PCB101 | 2,2',4,5,5'-pentaCB | 2.9% | <LOD | 24.2 | 3.8 |
| PCB110 | 2,3,3',4',6-pentaCB | 1.0% | <LOD | 12.6 | 2.0 |
| PCB128 | 2,2',3,3',4,4'-hexaCB | 1.0% | <LOD | 10.3 | 1.6 |
| PCB149 | 2,2',3,4',5',6-hexaCB | 2.9% | <LOD | 13.2 | 2.1 |
| PCB151 | 2,2',3,5,5',6-hexaCB | 1.0% | <LOD | 9.8 | 1.5 |
| PCB157 | 2,3,3',4,4',5'-hexaCB | 20.6% | <LOD | 32.4 | 7.6 |
| PCB167 | 2,3',4,4',5,5'-hexaCB | 20.6% | <LOD | 41.6 | 10.3 |
| PCB172 | 2,2',3,3',4,5,5'-heptaCB | 18.6% | <LOD | 12.8 | 2.3 |
| PCB177 | 2,2',3,3',4',5,6-heptaCB | 41.2% | <LOD | 17.5 | 3.8 |
| PCB178 | 2,2',3,3',5,5',6-heptaCB | 34.3% | <LOD | 15.0 | 3.1 |
| PCB189 | 2,3,3’,4,4’,5,5’-heptaCB | 4.9% | <LOD | 4.7 | 0.8 |
| PCB195 | 2,2',3,3',4,4',5,6-octaCB | 23.5% | <LOD | 9.7 | 1.4 |
| PCB206 | 2,2',3.3',4,4',5,5',6-nonaCB | 44.1% | <LOD | 17.0 | 2.5 |
| PCB209 | decaCB | 19.6% | <LOD | 42.0 | 10.4 |
| **Persistent pesticides** | |  |  |  |  |
| G-HCCH | γ-Hexachlorocyclohexane (Lindane) | 1.0% | <LOD | 16.6 | 3.2 |
| OP-DDT | 2-(4-chlorophenyl)-2-(2-chlorophenyl)- 1, 1, 1-trichloroethan | 2.9% | <LOD | 24.4 | 4.7 |
| MIREX | Mirex | 9.8% | <LOD | 524.4 | 55.3 |
| **Perfluorinated compounds (concentrations in μg/L)** | |  |  |  |  |
| Et-PFOsA-AcOH | 2-(N-ethyl-perfluorooctane sulfonamido) acetate | 2.0% | <LOD | 0.3 |  |
| Me-PFOSA-AC-OH | 2-(N-methyl-perfluorooctane sulfonamido) acetate | 51.0% | <LOD | 1.7 |  |
| PFDeA | perfluorodecanoate | 43.1% | <LOD | 1.0 |  |
| PFOSA | perfluorooctane sulfonamide | 0.0% | <LOD | <LOD |  |
